# Occult hepatitis B infection in chronic hemodialysis patients: Comparison of results and concepts: Occult HBV in chronic hemodialysis patients

**Published:** 2011-02-01

**Authors:** Amitis Ramezani, Mohammad Banifazl, Arezoo Aghakhani

**Affiliations:** 1 Clinical Research Department, Pasteur Institute of Iran, Tehran, IR Iran; 2 Iranian Society for Support Patients with Infectious Diseases, Tehran, IR Iran

**Keywords:** Occult hepatitis B infection, Hemodialysis

Dear Editor,

We greatly enjoyed reading the excellent review by Hollinger and colleagues on occult hepatitis B infection in chronic hemodialysis (HD) patients [1]. As Hollinger et al. mentioned in their review, few studies have shown the prevalence of occult hepatitis B virus (HBV) infection in HD patients. Due to the parenteral transmission of HBV, HD patients are at high risk of acquiring this virus because they need frequent blood transfusions and undergo medical procedures that accompany bleeding [2]. The prevalence of occult HBV infection in these patients is between 0 to 58% [3][4][5]. The different frequencies of occult HBV infection in HD patients may be due to variation in the prevalence of HBV infection among countries, sensitivity of molecular techniques, and the size and virological features of the study groups. In one study, we determined the rate of occult HBV infection in Iranian HD patients with isolated hepatitis B core antibody (anti-HBc) [6]. Of 289 patients enrolled in this study, 18 subjects had isolated anti-HBc and HBV-DNA was detected in 50% of patients who had isolated anti-HBc. Plasma HBV-DNA load was less than 50 IU/ml in each patient. Our investigation showed that occult HBV infection was common in HD patients with isolated anti-HBc regardless of age, sex, aminotransferases levels, or length of time on dialysis [6]. Our data is in agreement with the results of hollinger et al. [1] and suggests a relatively high prevalence of occult HBV infection in hemodialysis patients. Our study showed that detection of isolated anti-HBc could reflect unrecognized occult HBV infection in HD patients which is in concordance with the conclusions of Hollinger et al. The majority of these infections were associated with low viral loads [6]. In another study, we determined the genotype and surface gene mutations of HBV in HD patients with occult HBV infection. All HBV isolates belonged to genotype D. The most important mutations were the insertion of a single nucleotide, premature stop codons at Leu15, and Gly10 and S207N mutations. No "a" determinant mutations were found. Our study suggested that "a" regionmutations do not play a major role in HBsAg detection [7]. This review provides important information about the current concepts and strategy in occult HBV infection in chronic HD patients. Our study is limited in that we report no data on liver histopathology or outcome measurements; these should be investigated in the future.
